# Neurologists' detection and recognition of mental disorder in a tertiary in-patient neurological unit

**DOI:** 10.1192/bjb.2017.7

**Published:** 2018-02

**Authors:** Samr Dawood, Norman Poole, Robert Fung, Niruj Agrawal

**Affiliations:** 1 Springfield University Hospital, South West London and St George's Mental Health NHS Trust, London; 2 St George's Hospital, London

## Abstract

**Aims and method:**

Psychiatric disorders are common in neurological in-patients, but they are under-recognised and undertreated. We investigated the frequency of detection of mental disorder and referral to psychiatric services in a regional neuroscience centre. The results were compared with the expected prevalence. All in-patient referrals received in 2014 from the in-patient wards of the regional neuroscience centre and acute neurological unit were reviewed.

**Results:**

A total of 129 ward referrals were identified; of these, 78 were from the regional in-patient neurological unit, which comprised 11.4% of the total of 679 admissions to that unit.

**Clinical implications:**

A spectrum of neuropsychiatric conditions were recognised by neurologists, but overall rates of recognition were low. To address the problem of under-recognition, routine screening with validated assessment tools can represent a cost-effective and acceptable method to detect psychiatric disorders in an in-patient neurological setting.

**Declaration of Interest:**

None.

Liaison neuropsychiatry is an important element of psychiatric care in neurological in-patients, given the severity and complexity of the cases involved.^1^ Yet there is a dearth of information about consultation rates, presentation, diagnoses, and interventions that would improve access to services, facilitate evidence-based practice, and articulate the relationship between neurological and psychiatric disorders.^2^^–^^4^

Previous estimates of psychiatric comorbidity in neurological settings have revealed high prevalence rates. For example, the overall rate of psychiatric disorder in neurological out-patient clinics has been reported as 55.1%,^5^ the most frequent diagnoses being somatoform disorders, which represented 33.8% of the sample. Similarly, the prevalence in an acute in-patient neurological unit was 51.3%.^6^ It appears, therefore, that psychiatric comorbidity is high in neurological settings. This is to be expected, given the elevated rates of psychiatric comorbidity in neurological disorders. Patients with cerebrovascular and Parkinson's disease have prevalence of depression that ranges between 20 and 40%.^7^^,^^8^ Functional neurological symptoms are also common in neuroscience settings; it has been reported that 14% of consecutive neurological admissions had no so-called ‘organic’ bases for their symptoms, while another 24% had symptoms not fully accounted for by the underlying pathology.^9^

Neuropsychiatric conditions in neurological in-patients, if undetected or not adequately managed, are associated with poor quality of life, greater morbidity, mortality and poor psychosocial outcomes.^10^ Moreover, there are specific neuropsychiatric presentations associated with common neurological conditions that pose diagnostic, nosological and management challenges, which require the unique clinical skills of neuropsychiatrists. These conditions may include schizophrenia-like psychosis of epilepsy, forced normalisation,^11^ organic personality changes or organic mood disorders,^12^ to name a few. This highlights the need for the development and implementation of structured care pathways for the neuropsychiatric comorbidities associated with neurological conditions.^13^

Despite these high rates of psychiatric comorbidity, surveys investigating referrals from neurology wards to neuropsychiatric liaison services have demonstrated unexpectedly low rates of referral. Fitzgerald *et al* and Jonge *et al* found that only 6% and 2.4% of neurology in-patients, respectively, were referred to liaison neuropsychiatry.^14^^,^^15^ The present study sought to investigate the rates of referral to an established neuropsychiatry service in a tertiary neurosciences centre, in order to better understand referral patterns and rates of recognition of mental disorder in neurological in-patients.

## Method

This study was conducted at the Atkinson Morley Regional Neurosciences Centre at St George's Hospital, London, covering a population of 3 million residents of South West London and Surrey. All referrals made to the neuropsychiatry service at St George's Hospital over a 12-month period in 2014 were included.

We reviewed the referral forms and electronic patient records to extract information on demographics, reasons for referral, diagnoses, treatment plan, and number of contacts made. We identified the reasons for referral from the free text provided by the referrers. This requested information gives specific details about reasons for referral, presenting symptomology, referrers’ suspected diagnoses, previous psychiatric history and interventions, and management difficulties. Reasons for referral were grouped into categories, and more than one reason for each patient was allowed. The patient electronic records system used to record every contact with patients was also searched to retrieve the primary ICD-10 diagnoses for each patient. These results were then compared against those available in the published literature on neuropsychiatry and adult liaison psychiatry services referral patterns.

Those referred to the neuropsychiatry service from the acute neurology ward were compared against admission data for that ward to establish rates of referral. This was then contrasted with results from previous studies of this ward investigating the prevalence and rate of detection of mental disorder by neurologists. All clinicians involved in this study work within the clinical team and had access to patients' records as part of their role. Anonymised data were analysed and compared with previously published data.

## Results

A total of 129 referrals were identified in the year 2014. They were evenly dispersed across age groups, with a small peak in the age group 50–59. The female:male ratio for referral was 1.35:1 (*P* = 0.09). Almost 50% of the sample had a past psychiatric history. In terms of face-to-face contacts made, 32% received initial assessment only, 27% were seen twice, 15% were seen three times, and 10% were seen four times. The remaining 16% had four or more face-to-face contacts. The cumulative sum of all face-to-face contacts (first assessment and follow-up) was 311. Usually, the first assessments lasted 45–75 minutes and 90% were seen within 2 working days of the date of referral. The highest rates of referral were during the months March, June, October and December.

Sixty per cent of referrals came from the regional in-patient neurology ward, 15% from neurosurgical wards, 9% from the stroke units, and 4% from the neuro-intensive care unit (NICU). The remainder were referred from other wards. Only 7% were made as urgent referrals. It was documented on 75% of forms that the referrer had discussed referral with the patient.

**Fig. 1:**
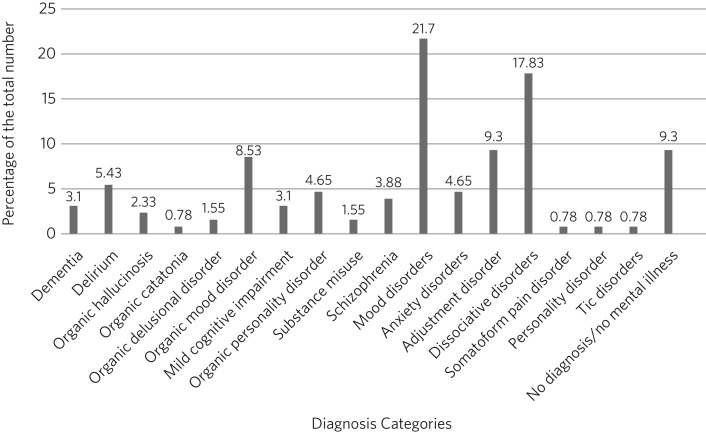
Distribution of patients per diagnostic categories.

The most frequent cited reasons for referral were depression (50%), functional neurological symptoms or functional overlay (27%), anxiety (22%), cognitive decline or confusion (17%), agitation/aggression (13%), suicidal ideation or behaviour (12%), and psychotic symptoms (12%). Often, more than one reason for referral was provided; hence, the percentages do not total 100%. As a reason for referral, ‘agitation’ was associated most with organic disorders, ‘suicidal’ with adjustment disorder and organic mood disorder, and ‘depression’ with mood disorders, adjustment disorder and ‘no diagnosis’. Functional symptoms/overlay were invariably associated with a psychiatric diagnosis of dissociative/conversion disorders. Almost all (91%) of the referred patients met the criteria for a psychiatric disorder according to ICD-10. The most common primary psychiatric diagnoses were: mood disorder (22%); dissociative disorder (18%); adjustment disorder (9%); delirium (5%); organic disorders (24%), including organic mood disorder (8.5%); and organic personality disorder (5%). In 9% of those referred, no mental disorder was established (Fig. 1).

Seventy-eight of the referrals were sent from the regional in-patient neurological unit, comprising 11% of the total of 679 admissions to that unit. The age of referred patients was distributed evenly across the decades of life, with a small peak in the 20–29 age group. The female:male ratio was 1.4:1. Initial assessment only was completed for 31%, while 27% were seen twice, 14% three times, and the remainder four times or more. Urgent referrals constituted 10% of all referrals from this ward, and 83% of referral forms had documented a discussion with the patient. Collectively, depression, anxiety and adjustment disorders represented the most frequent diagnoses (32%), followed by ‘organic’ disorders (31%) and then somatoform disorders (21%). Most of those referred (81%) had a past history of psychiatric disorder. The majority of those patients diagnosed with dissociative disorders (70%) attended out-patient follow-up with our service following discharge.

## Discussion

This is a retrospective study of referrals from a regional neurology unit, which can come with its own limitations. Such a unit would generally have more complex neurological in-patients with a higher rate of neuropsychiatric comorbidity. However, the results of the present study and previously published papers are broadly comparable, suggesting no specific biases associated with our study population. The total number of referrals was small, despite the expected prevalence of psychiatric illnesses in neurological settings. This is in keeping with the low rates of identification of psychiatric comorbidity found in earlier prevalence studies. It may also suggest that neurology colleagues have a high threshold for referral to the psychiatric services. However, this implies that patients suspected to have psychiatric disorder were not referred to specialist services.

Our results show that patients referred to the team presented with a wide range of neuropsychiatric disorders (Fig. 1). The rates of mental disorder in our sample did not match those in community^16^ or general hospital^17^ settings, demonstrating the distinctive nature of neuropsychiatric samples. The most common categories in our population were mood disorders, organic disorders and dissociative disorders, while the most common reasons for referrals were ‘depression’ and ‘functional neurological symptoms’. Rates of disorder in our sample were close to those reported in previous studies,^14^^,^^15^ with a few differences.^17^ One difference is that depressive disorder accounted for 40% of referrals reported by Guthrie *et al* to their general liaison service that serves a large teaching hospital, but only 20% of our referrals received this diagnosis. Nevertheless, as for Guthrie *et al*, ‘depression’ was the leading reason for referral to our service and the most common diagnosis in our sample. Schizophrenia and bipolar disorders constituted a small portion of our workload, while they comprised one-third for Guthrie *et al*. Other significant differences were found in the rates of somatoform disorders (18.6 *v.* 2.6), organic disorders (24 *v.* 1.7), substance use disorders (4.8 *v.* 1.55) and personality disorders (0.7 *v.* 3.8), as shown in Table 1. Not unexpectedly, perhaps, our neuropsychiatry team was referred a higher proportion of organic disorders compared with the general hospital liaison service of Guthrie *et al*. It might be that neurologists perceive neuropsychiatrists as more willing and/or able to manage patients with organic psychiatric disorders than a liaison psychiatric service, such as that of Fitzgerald *et al*. Alternatively, because the Atkinson Morley Centre is a tertiary unit which admits complex neurological cases, there may be a higher percentage of organic psychiatric disorder in the population we serve. Another difference was the higher rate of dissociative disorders compared with liaison psychiatry teams, which again likely reflects that a tertiary neurology centre admits the most complex functional cases for thorough investigation and intervention – especially as there is a dearth of neuropsychiatry services outside the London area. The unexpectedly low rate of substances misuse and personality disorders identified in our sample could reflect a reluctance to discuss these issues or an oddity of our population. Either way, this finding merits further evaluation. These variations will differentially influence the clinical expertise and practice of neuropsychiatrists and their colleagues in liaison psychiatry.

**Table 1:** Comparison of psychiatric diagnoses as a proportion of total number of referrals (%)

Diagnostic categories	Jonge *et al*, 2001 (neurological ward referrals to general liaison psychiatric service)	Fitzgerald *et al*, 2008 (neurological ward referrals to general liaison psychiatric service)	Dawood *et al*, 2016 (neurological ward referrals to neuropsychiatric service)	Guthrie *et al*, 2016 (general non-neurological ward referrals to general liaison psychiatric service)
Diagnostic system	ICD-10	DSM-IV	ICD-10	ICD-10
Mood disorders	15.1	24	21.7	46.5
Somatoform disorder/dissociative disorders/ Medically unexplained symptoms	19.3	23	18.6	2.6
Anxiety disorders/adjustment disorders	15.5	11	12.2	6.4
Organic disorders, including dementia	16	5	24	5.2
Delirium	3.8		5.4	6.7
Substance use disorders	4.4	20	1.55	4.7
Psychosis/schizophrenia	2.7	5	3.9	14.8
Personality disorders			0.77	3.8
Others	7.9		2.33	2
No mental illnesses/differed	15.1	12	9.3	7.3
Total	100	100	100	100

Regarding the acute in-patient neurological unit, our results demonstrated a referral rate of only 11.16%, although previous studies on the same ward have demonstrated substantially higher prevalence rates. Utilising a battery of screening questionnaires followed by psychiatric interview Jeffries *et al*^6^ identified a DSM-IV-defined mental disorder in 51.3% of 265 consecutive admissions during a period of 6 months. Of these, 18.7% fulfilled the criteria for two diagnoses, and 5.1% were diagnosed with three or more. Earls *et al*^18^ investigated rates of detection of psychiatric symptoms by neurologists on this same ward 3 months pre and post Jeffries *et al*'s screening period. This showed that neurologists recognised and documented symptoms of mental illnesses in 23.7% of all admissions, but referred fewer than half of these (10.4%), echoing our more recent findings (11.6%). Taken together, this demonstrates that 70–80% of neurological patients with a comorbid psychiatric disorder are not being referred to specialist services. Given the known impact of neuropsychiatric comorbidity on quality of life, duration of hospital stay, mortality, and cost of care, this may have a deleterious effect on those unable to access timely and effective psychiatric intervention.^19^ Of those who were referred, analysis of the reason(s) for referral indicated strong correlation with the eventual confirmed diagnosis. The small number of patients who did not receive any diagnosis points to a low rate of false positives. Thus, it appears that neurology referrers were specific but not sensitive to identification of cases. Table 2 suggests that few patients with anxiety, adjustment disorders, personality disorders, and substance misuse disorders were referred, while referral was made for only a minority of those with depression and cognitive disorders. Appropriately, all those with psychotic disorders were referred, as were half the patients with somatoform disorders. Regarding patients with cognitive impairment, it may be that neurologists consider themselves capable of managing this patient group, as agitation was given as the main reason for the referral in all cases involving that problem.

**Table 2:** Comparison of psychiatric diagnoses/symptoms as a proportion of total admissions in the specific neurological in-patient unit (%)

Diagnostic categories	Jefferies *et al*, 2007	Earl *et al*, 2011	Dawood *et al*, 2016
Diagnostic system	Prevalence of psychiatric diagnoses DSM-IV	Psychiatric symptoms / problems detected by neurologist	Diagnoses referred by neurologists, ICD-10
Mood disorders plus organic mood disorders	24.8	9.2	3
Delirium, dementia and cognitive disorders	17.7	6.7	0.88
Anxiety	12.7	2.2	0.73
Adjustment disorders	4.6	0	0.88
Somatoform disorders	4.5	6.4	2.5
Substance use disorders	3		0.1
Personality disorders	2		0.29
Disorders usually diagnosed in childhood	2		0.1
Other disorders that may be of clinical importance	2		0
Psychotic disorders	1	1.6	1
Eating disorders	0.5		0
Other organic disorders			1.1
No diagnosis			0.58
Total^a^	51.1	23.7	11.16

a. Percentages add up to more than total because some cases had two or more comorbid psychiatric diagnoses.

It is unclear how this under-referral affects the well-being of patients and the efficiency of neurological departments. Similarly, Jonge *et al* found that neurologists throughout Europe refer only a small proportion of the psychiatric patients on their wards. Possibly, their recognition of mental disorder is poor, or these comorbidities are considered irrelevant to their neurological care. Jonge suggested a referral procedure consisting of a short questionnaire to facilitate detection of caseness.^15^ Likewise, Jeffries *et al* concluded that psychiatric screening questionnaires have a high sensitivity and specificity, thereby representing a cost-effective and acceptable method for improving identification of psychiatric morbidity and comorbidity.^6^ The intervening years have not lessened the arguments for this approach. There is now a pressing need for strategic planning to develop neuropsychiatric provision, both nationally and internationally.^20^^–^^22^ Provision of prospective screening on neurological units and the impact of neuropsychiatric input would require prospective evaluation to evaluate their utility and efficacy.

## About the authors

**Dr Samr Dawood**, MBCHB, FICMS, MRCPsych, Higher Trainee in General Adult Psychiatry, Springfield University Hospital, South West London and St George's Mental Health NHS Trust, London, UK; **Dr Norman Poole**, MD, MRCPsych, MSc, Consultant Neuropsychiatrist, St George's Hospital, London, UK; **Dr Robert Jr Fung**, MRCPsych, Speciality Doctor in Neuropsychiatry in South West London and St George's Mental Health NHS Trust, St George's Hospital, London, UK; **Dr Niruj Agrawal**, MBBS, MD, MSc, Dip CBT, FRCPsych, Consultant Neuropsychiatrist and Honorary Senior Lecturer, St George's Hospital, London, UK.
